# Emergency surgeon: “ last of the mohicans” 2014-2016 editorial policy WSES- WJES: position papers, guidelines, courses, books and original research; from WJES impact factor to WSES congress impact factor

**DOI:** 10.1186/1749-7922-9-14

**Published:** 2014-02-03

**Authors:** Fausto Catena, Frederick Moore, Luca Ansaloni, Ari Leppäniemi, Massimo Sartelli, Andrew B Peitzmann, Walt Biffl, Federico Coccolini, Salomone Di Saverio, Belinda De Simone, Michele Pisano, Ernest E Moore

**Affiliations:** 1 Emergency Surgery, Maggiore Parma Hospital, Parma, Italy; 2 Department of Surgery, University of Florida, Gainesville, FL, USA; 3 General Surgery Department, Papa Giovanni XXIII hospital, Bergamo, Italy; 4 Department of Abdominal Surgery, University Hospital Meilahti, Helsinki, Finland; 5 Department of Surgery, Macerata Hospital, Macerata, Italy; 6 Department of Surgery, University of Pittsburgh School of Medicine, Pittsburgh, PA, USA; 7 Department of Surgery, Denver Health Medical Center, Denver, CO, USA; 8 Department of Surgery, Maggiore Hospital, Bologna, Italy

## 

At the WSES Bergamo Congress last July the WSES- WJES board established the Scientific Development Policy (SDP) for the Society and Journal for the next 2 years (2014-2016).

The project is based on the idea to create an organized scientific movement having the objective to standardize the state of the art for emergency surgery, while attempting to develop guidelines for related topics and promoting original research.

The first aim of this the strategy is to start with a careful analysis of existing literature leading to the creation of position papers. (SDP first step).

Generally a member of WSES- WJES Board (after agreement of the WSES Board of Directors - WJES Editorial Board) is charged to perform this first SDP step.

The position paper is then published in the WJES, and the following two years Consensus Conferences are scheduled to prepare guidelines that will be presented at the ensuing WSES World Congress.

After the World Congress these guidelines will be published in the WJES.

During these 3 years original research is encouraged to clarify these defined topics.

The idea to create a scientific virtuous cycle with the ultimate goal to define the evidence based literature and stimulating research to give emergency surgeons useful tools.

Globally the huge rise in claims by patients, the increase in operating costs of the facilities in which the medical service is rendered and the increasingly important role of insurance have pushed the various levels of government authorities (local health care agencies and hospitals, Regional Governments and National Health Ministry) to implement control systems and risk prevention organizations during the performance of therapeutic activities.

Actually a decisive role in the organization and management of health facilities is played by the risk management although the classification of errors in health care represents a complex task under different points of views. In the risk management evaluation has always to be pointed out the specificity of individual patients, the risk of some types of procedures with the multiplicity of professional experiences and the range of management models of the various health care facilities. In the prevention of clinical risks, although attention has focused primarily on improving the knowledge and training of the individual practitioner, however it has been noted that often the error, rather than depend on the conduct of the health professional, is the result of objective shortcomings organizational structures themselves. In this arrangement, a central role is taken by the clinical guidelines that are usually prepared by scientific societies and, on the basis of Evidence Based Medicine, may be recognized as real rules of professional conduct and certified practices to which the professionals and the hospitals must follow. These recommendations regarding the practical clinical behavior are based on the latest scientific studies and may come directly or indirectly from public and private organizations, national or international.

In general the use of guidelines as a criterion for identifying the responsibility of the physician has long been used by law prone to assess the legitimacy of the behavior of health professional as the compliance with “good clinical practice”, without thereby hiding the limits that this criterion in itself entails. Guidelines are not, in fact, mandatory rules in absolute terms, but general guiding principles and sometimes a bit theoretical, that may become soon obsolete due to the rapid and steady progress of science and relatively inapplicable due to the margins of unpredictability of the medicine related to the concrete individual case.

The risk of guidelines is to reduce the freedom of action of the health professional and constrain the choices at the expense of possible alternative solutions, eventually still effective and even more beneficial to scientific progress. In fact the surgeon, who in the daily practice is limited to adhere to the guidelines, inevitably produces an arrest of the evolution of scientific thought and of clinical trials.

While bearing in mind these limitations and disadvantages of the guidelines is important that the scientific societies provide for their construction and updates to help the surgeons in their common daily practice.

This tool is especially useful in emergency and trauma surgery where treatment decisions are to be taken in times that are not compatible with the usual scientific update.

For these reasons, the WSES has placed (and will continue to place) a lot of effort in building guidelines that involve as much as possible professionals working in different countries and continents in order to provide common tools to identify the best clinical practice.

The dissemination of guidelines and recommendations is perhaps even a more important activity than their creation.

Is therefore intentional and wanted the increased publication of WSES guidelines on WJES. We hope that these tools will be useful and appreciated by the emergency and trauma surgeons from around the world.

After obtaining an impact factor for WJES, our next new challenge is to develop a WSES Congress impact factor based on the quality of the Congress and on its intrinsic capacity to support SDP virtuous cycle.

The “WJES- WSES impact factor Task Force” has developed the mathematical formula to be applied on the last WSES Congress.

The WSES-WJES Educational Team has also recently completed the first educational project with the issuing of the WSES Trauma Surgery Book. The main aim of these two volumes is to provide a fresh view of the surgical approach to trauma patients, by mean of practical suggestions, surgical techniques and organizational issues for improving the skills of trainee surgeons as well as anyone who is dealing with trauma patients. The worldwide contribution to these books is evident by the participation of trauma professionals from five continents and the coverage of multidisciplinary topic, across several surgical and critical care subspecialties (Volume 1 covers Trauma Management, Trauma Critical Care, Orthopaedic Trauma and Neuro-Trauma [1], Volume 2 focuses on Thoracic and Abdominal Trauma [2]. The books aim to purposely fall within the multidisciplinary educational scope of our SDP planned by a truly “World” Society of Emergency Surgery.

The next steps of this project on education of the Emergency Surgeon worldwide will be an Acute Care (non-Trauma) Surgery book and WSES-WJES Courses, which will promulgate emergency surgery education around the world, by using WSES- WJES guidelines.

In our era we have observed the onset of many general surgery subspecialization: (minimal invasive, bariatric,, upper GI, HBP, colorectal etc…).

In this context emergency surgeons appears to remain the “last general surgeons” able to perform a emergency thoracotomy or a liver resection after a DCS for trauma [2-7].

We are probably the “last of the Mohicans” but the need for these skill sets is increasing. The WSES- WJES mission is to support this expertise, aiming to promulgate the information globally.

The WSES – WJES education program (including up-to-date books and surgical courses including hands-on sessions) is critical for this mission.
