# Correction: emergency surgeon: “last of the mohicans” 2014–2016 editorial policy WSES-WJES: position papers, guidelines, courses, books and original research; from WJES impact factor to WSES congress impact factor

**DOI:** 10.1186/1749-7922-9-25

**Published:** 2014-04-03

**Authors:** Fausto Catena, Frederick Moore, Luca Ansaloni, Ari Leppäniemi, Massimo Sartelli, Andrew B Peitzmann, Walt Biffl, Frederico Coccolini, Salomone Di Saverio, Belinda De Simone, Michele Pisano, Ernest E Moore

**Affiliations:** 1 Emergency Surgery, Maggiore Parma Hospital, Parma, Italy; 2 Department of Surgery, University of Florida, Gainesville, FL, USA; 3 General Surgery Department, Papa Giovanni XXIII hospital, Bergamo, Italy; 4 Department of Abdominal Surgery, University Hospital Meilahti, Helsinki, Finland; 5 Department of Surgery, Macerata Hospital, Macerata, Italy; 6 Department of Surgery, University of Pittsburgh School of Medicine, Pittsburgh, PA, USA; 7 Department of Surgery, Denver Health Medical Center, Denver, CO, USA; 8 Department of Surgery, Maggiore Hospital, Bologna, Italy

## Correction

Since the publication of our Commentary [1], we noticed that Dr. Salomone Di Saverio was incorrectly excluded from and the titles of the books and the order of authors was incorrect in References 1 and 2. The correct references are:

1. Di Saverio S, Tugnoli G, Catena F, Catena G, Ansaloni F, Naidoo N: *Trauma Surgery Volume 1: Trauma Management, Trauma Critical Care, Orthopaedic Trauma and Neuro-Trauma*. Verlag Italy: Springer; 2014. ISBN 978-88-470-5403-5.

2. Di Saverio S, Tugnoli G, Catena F, Catena G, Ansaloni F, Naidoo N: *Trauma Surgery Volume 2: Thoracic and Abdominal Trauma*. Verlag Italy: Springer; 2014. ISBN 978-88-470-5459-2.
